# Interaction between smoking and HLA-A*02:01 in multiple sclerosis progression

**DOI:** 10.1177/13524585261453243

**Published:** 2026-07-03

**Authors:** Anna Karin Hedström, Pernilla Stridh, Jan Hillert, Tomas Olsson, Lars Alfredsson

**Affiliations:** Department of Clinical Neuroscience, Karolinska Institute, Stockholm, Sweden; Department of Clinical Neuroscience, Karolinska Institute, Stockholm, Sweden; Department of Clinical Neuroscience, Karolinska Institute, Stockholm, Sweden; Department of Clinical Neuroscience, Karolinska Institute, Stockholm, Sweden; Institute of Environmental Medicine, Karolinska Institute, Stockholm, Sweden; Centre for Occupational and Environmental Medicine, Region Stockholm, Stockholm, Sweden

**Keywords:** Multiple sclerosis, human leukocyte antigen, smoking, disability progression, gene–environment interaction

## Abstract

**Background:**

Gene–environment interactions between smoking and HLA genotypes have been reported in multiple sclerosis (MS) susceptibility, although their relevance beyond disease onset remains unclear.

**Objectives:**

To examine whether the effect of smoking on disability progression differs according to HLA-A*02:01 and HLA-DRB1*15:01 status.

**Methods:**

Patients from two population-based case–control cohorts were classified by smoking status at diagnosis and by HLA-A*02:01 and HLA-DRB1*15:01 status and were followed for up to 15 years through the Swedish MS registry (*n* = 6807). Cox regression models were used to estimate hazard ratios (HRs) with 95% confidence intervals (CIs) for 24-week confirmed disability worsening (CDW) and time to Expanded Disability Status Scale (EDSS) 3, 4, and 6. Interaction was assessed on the additive scale.

**Results:**

Compared with HLA-A*02:01-positive non-smokers, smoking at diagnosis was associated with a higher risk of disability progression primarily among individuals who lacked HLA-A*02:01, with consistently elevated risks of CDW (HR = 1.15, 95% CI = 1.06–1.26), EDSS 3 (HR = 1.26, 95% CI = 1.08–1.47), and EDSS 4 (HR = 1.50, 95% CI = 1.25–1.79). No clear effect of smoking was observed among HLA-A*02:01 carriers, and no evidence of interaction was detected between smoking and HLA-DRB1*15:01.

**Conclusion:**

These findings suggest that the impact of smoking on MS progression varies according to immunogenetic background.

## Background

Multiple sclerosis (MS) is a chronic inflammatory and neurodegenerative disease with considerable heterogeneity in clinical course. While the etiology of MS is strongly influenced by the interplay between genetic and environmental risk factors,^1–3^ the determinants of disability progression after onset remain less well understood.

Smoking is one of the most consistently replicated environmental risk factors for MS,^4^ and growing evidence indicates that it also accelerates disability accumulation after diagnosis.^5–7^ Proposed mechanisms include chronic systemic inflammation, oxidative stress, and enhanced microglial activation, all of which may exacerbate neurodegenerative processes.^6,7^

Genetic influences on progression appear limited. Only one genome-wide association study has identified a significant locus for MS severity, with modest effect on disability progression,^8^ and MS polygenic risk scores show limited relevance for post-onset disease course.^9^ Nevertheless, specific genetic backgrounds may modify the effects of environmental exposures, even if they do not exert strong overall effects.

Smoking has been found to strongly interact with key human leukocyte antigen (HLA) risk genotypes (HLA-DRB1*15:01 positivity and absence of HLA-A*02:01), resulting in synergistic increases in susceptibility.^10^ Given the biological plausibility of such interactions and their magnitude for risk,^1–3^ similar gene–environment interplay may also influence disability progression, particularly through pathways linked to neurodegeneration and chronic immune activation. Yet this possibility has rarely been studied.

Using a large, population-based cohort with longitudinal disability follow-up, we, therefore, investigated whether smoking-related disability progression varies by HLA-DRB1*15:01 or HLA-A*02:01 status.

## Methods

Our study comprised patients from two nationwide population-based case–control studies: EIMS (Epidemiologic Investigation of Multiple Sclerosis)^11^ and GEMS (Genes and Environment in Multiple Sclerosis).^11^ Incidence cases of MS were recruited to EIMS through hospital-based neurology units between April 2005 and December 2015 (*n* = 2424), whereas GEMS identified prevalent cases, distinct from those in EIMS, from the Swedish MS registry between November 2009 and November 2011 (*n* = 6148). Eligibility criteria for cases were age 16–70 years, residence in Sweden, and a neurologist-confirmed diagnosis of MS according to the McDonald criteria.^12^ The response rate was 93% in EIMS and 82% in GEMS.

All participants completed a standardized questionnaire at study inclusion and were invited to provide blood samples for genetic analyses. HLA data were available for 6807 (79%) of the 8572 participants, all of whom were included in this study (Supplemental eFigure 1).

The studies were approved by the Regional Ethical Review Board at Karolinska Institute and conducted in accordance with the ethical standards of the 1964 Declaration of Helsinki and its later amendments. All participants provided written informed consent.

## Definition of exposures

Information on environmental exposures and lifestyle habits was collected at study inclusion. Smoking habits were assessed through questions on current and previous smoking, including duration (time periods) and intensity (average number of cigarettes smoked per day for each time period). Smoking was categorized as current smoking at diagnosis versus non-smoking. We focused on current smoking at diagnosis, as previous studies indicate that ongoing smoking has the strongest association with MS progression, whereas past smoking alone does not appear to influence disability worsening.^7,13^

Ancestry was based on the self-reported country of birth of the participant and their parents. Individuals were classified as having Nordic ancestry if they and both parents were born in a Nordic country.

HLA alleles were identified at two-field resolution. Genotyping was conducted using the MS replication chip,^14^ and HLA alleles were imputed using HLA*IMP:02 based on a multi-population reference panel.^15^ Participants were categorized according to the presence or absence of HLA-DRB1*15:01 and HLA-A*02:01. These alleles were selected since they form the key HLA risk background shown to interact with smoking in MS susceptibility.^10^

## Outcome measures

The Swedish MS registry is used in all neurology units nationwide and is integrated into the clinical documentation system.^16^ For each patient, healthcare professionals prospectively record detailed information on medical treatments, disease activity, and physical functioning.

To analyze disability progression, the baseline was defined as the date of the first recorded EDSS score. Confirmed disability worsening (CDW) was defined as an increase in EDSS sustained across two visits at least 6 months apart: ⩾1.5 points if baseline EDSS was 0, ⩾1 point if baseline EDSS was between 1.0 and 5.0, and ⩾0.5 points if baseline EDSS was ⩾5.5. Analyses of time to reach EDSS milestones scores of 3, 4, and 6, confirmed at two consecutive visits, were limited to patients with a baseline EDSS score below 3.

## Statistical analysis

Differences in baseline characteristics between groups were assessed using Student’s *t*-tests for continuous variables and chi-square tests for categorical variables. Time-to-event analyses were conducted using Cox proportional hazard regression to estimate hazard ratios (HRs) with 95% confidence intervals (CIs) for disability progression. Participants were followed from the baseline until the onset of the events of interest, loss to follow-up, death, or end of study (6 April 2022), whichever occurred first. The proportional hazard assumption was tested through the Schoenfeld residuals, with no violations of proportionality observed. Analyses were adjusted for age at baseline, sex, ancestry, past IM, calendar year of diagnosis, disease phenotype (relapsing–remitting MS, secondary progressive MS, primary progressive MS, or unknown), disease duration at baseline, baseline EDSS, and disease-modifying therapy (DMT) exposure, summarized as the proportion of the duration of follow-up spent on disease-modifying therapy.

We examined whether smoking intensity and cumulative smoking at diagnosis were associated with disability progression within strata of HLA-A*02:01 and HLA-DRB1*15:01. Smoking intensity was categorized into <1, 1–10, 11–20, and >20 cigarettes per day and cumulative smoking into 0–10, 11–20, and >20 pack-years. Analyses were performed separately for each genotype group defined by the presence or absence of HLA-A*02:01 or HLA-DRB1*15:01, using the same covariate adjustments as in the primary Cox proportional hazards models.

Interaction between smoking and HLA genotypes was assessed by creating four exposure groups using non-smokers with the relevant protective genotype (HLA-A*02:01-positive or HLA-DRB1*15:01-negative) as the reference category. Interaction on an additive scale was evaluated using the attributable proportion due to interaction (AP).^17^ Additive interaction was chosen since it provides the most interpretable measure of whether the combined effect of smoking and HLA genotype exceeds the sum of their individual effects. Confidence intervals for AP were derived using the delta method. Kaplan–Meier curves were generated for descriptive purposes.

### Sensitivity analyses

Several sensitivity analyses were conducted. We restricted analyses to participants of Nordic ancestry to reduce population stratification and minimize confounding related to ancestry-associated differences in HLA allele frequencies. Since DMT use strongly influences disease outcomes, we performed sub-analyses in which we excluded untreated participants and further adjusted for DMT exposure as a time-updated variable. Finally, we limited the analyses to incident MS cases with further adjustment for demographic and lifestyle covariates at the time of diagnosis: educational attainment (pre-secondary, secondary, or post-secondary education), body mass index (<25, 25–30, >30 kg/m^2^), alcohol consumption (no consumption, low, moderate, or high consumption according to cutoffs used by Statistics Sweden), fish consumption, sun exposure habits, and physical activity. Fish consumption was assessed with two 4-point items on the frequency of lean and oily fish intake. Scores were summed and dichotomized at the median (high vs low). Sun exposure was assessed with three 4-point items on ultraviolet radiation exposure. Scores were summed to an index of 3–12 and dichotomized at the median (high vs low). Physical activity at diagnosis was modeled as a four-level exposure (low, moderate, moderate–high, high). Additional analyses were performed using time to EDSS 6 as the outcome. Finally, ancestry-informative principal components derived from genome-wide genotype data were used to further adjust for population stratification. In this analysis, population outliers were excluded (*n* = 85). Analyses were conducted in SAS version 9.4.

## Results

A total of 6807 individuals with MS were included, of whom 2296 (34%) were smokers at diagnosis. The median follow-up time was 11.1 years (range: 7.2–16.3) with similar follow-up across combined smoking and HLA strata (Supplemental eTable 1). Smokers were slightly younger at disease onset, had a higher mean EDSS at baseline, and reported worse physical health-related quality of life, compared with non-smokers. Use of disease-modifying treatment (DMT) and time from onset to DMT initiation were similar between groups. The distribution of HLA-DRB1*15:01 and HLA-A*02:01 was also similar in smokers and non-smokers (Table 1).

**Table 1.** Characteristics of the overall sample and by smoking status at diagnosis.

		Total	Smokers	Non-smokers	*p*
*N*		6807	2296	4511	
Median follow-up (range)		11.1 (7.2–16.3)	11.4 (6.4–15.5)	10.9 (6.2–15.2)	
Age at disease onset (SD)		33.3 (10.6)	32.4 (10.3)	33.8 (10.7)	0.07
Age at diagnosis (SD)		37.8 (11.3)	36.0 (11.2)	38.7 (11.2)	<0.0001
Sex, *n* (%)	Female	4956 (72.8)	1653 (72.0)	3303 (73.2)	0.28
	Male	1851 (27.2)	643 (28.0)	1208 (26.8)	
Ancestry, *n* (%)	Nordic	5967 (87.7)	2002 (87.2)	3965 (87.9)	0.41
	Non-Nordic	840 (12.3)	294 (12.8)	546 (12.1)	
MS phenotype, *n* (%)	Relapsing-onset	6159 (90.5)	2091 (91.1)	4068 (90.2)	0.37
	Progressive-onset	569 (8.4)	180 (7.8)	389 (8.6)	
	Unknown	79 (1.2)	25 (1.1)	54 (1.2)	
DMT, *n* (%)	Never	1646 (24.2)	587 (25.6)	1059 (23.5)	0.06
	Platform	2831 (41.6)	960 (41.8)	1871 (41.5)	
	High-efficacy	1663 (24.4)	221 (9.6)	446 (9.9)	
	Escalation	667 (9.8)	528 (23.0)	1135 (25.2)	
Time to DMT initiation, years (SD)		2.2 (5.2)	2.4 (5.6)	2.7 (6.1)	0.06
Baseline EDSS (SD)		2.7 (2.0)	3.0 (2.1)	2.6 (2.0)	<0.0001
Baseline MSIS-PHYS (SD)		26.8 (23.5)	31.5 (24.8)	24.7 (22.6)	<0.0001
Past IM, *n* (%)	Yes	686 (13.8)	294 (12.8)	619 (13.7)	0.05
	No	3490 (70.4)	1566 (68.2)	3215 (71.3)	
	Unsure/missing	780 (15.7)	436 (19.0)	677 (15.0)	
HLA-A*02:01 status, *n* (%)	Positive	2911 (42.3)	1001 (43.6)	1910 (42.3)	0.32
	Negative	3896 (57.2)	1295 (56.4)	2601 (57.7)	
HLA-DRB1*15:01 status, *n* (%)	Positive	3904 (57.4)	1321 (57.5)	2583 (57.3)	0.83
	Negative	2903 (42.7)	975 (42.5)	1928 (42.7)	

DMT: disease-modifying therapy; EDSS: Expanded Disability Status Scale; SD: standard deviation; MSIS-PHYS: Multiple Sclerosis Impact Scale physical component; IM: infectious mononucleosis; HLA: human leukocyte antigen. Escalation DMT represents initiation with platform therapy and subsequent escalation to high-efficacy DMT.

Across HLA-A*02:01 strata, demographic and clinical characteristics were broadly similar, with small differences observed in ancestry distribution and baseline EDSS. Past IM was somewhat more common among HLA-A*02:01-negative participants, in line with known EBV-HLA relationships.^18^ Participants stratified by HLA-DRB1*15:01 status also showed largely comparable demographic and clinical profiles. DRB1*15:01-positive individuals were slightly younger, more often female, and more frequently of Nordic ancestry (Supplemental eTables 2 and 3).

Associations between smoking and disability outcomes were evaluated using Cox proportional hazards models. We first examined smoking intensity and cumulative smoking within strata of HLA genotype and subsequently evaluated the joint effects of smoking and HLA status using combined exposure categories to assess interaction. Smoking intensity and cumulative smoking were associated with an increased risk of CDW among HLA-A*02:01-negative individuals, but not among HLA-A*02:01-positive individuals. No effect modification was observed by HLA-DRB1*15:01 (Table 2).

**Table 2.** Trend associations between smoking intensity and cumulative smoking at diagnosis and confirmed disability progression, stratified by HLA-A*02:01 and HLA-DRB1*15:01 status.

Stratified by HLA-A*02:01 status					
A*02:01 status	*N*	Years (SD)	CDW (%)	Smoking intensity HR (95% CI)^1^	Cumulative smoking HR (95% CI)^2^
A*02:01 positive	2911	6.8 (5.1)	1815 (62.4)	1.02 (0.98–1.07)	1.03 (0.97–1.08)
A*02:01 negative	3896	6.7 (5.2)	2436 (62.5)	**1.06 (1.02–1.11)**	**1.08 (1.03–1.13)**
Stratified by HLA-DRB1*15:01 status					
DRB1*15:01 status	*N*	Years (SD)	CDW (%)	Smoking intensity HR (95% CI)^1^	Cumulative smoking HR (95% CI)^2^
DRB1*15:01 negative	2903	6.7 (5.1)	1818 (62.6)	**1.06 (1.02–1.12)**	**1.07 (1.01–1.13)**
DRB1*15:01 positive	3904	6.8 (5.2)	2433 (62.3)	**1.03 (1.00–1.07)**	**1.05 (1.00–1.10)**

HLA: human leukocyte antigen; CDW: confirmed disability worsening; HR: hazard ratio; CI: confidence interval; SD: standard deviation; EDSS: Expanded Disability Status Scale; DMT: disease-modifying therapy. Hazard ratios reflect the change in risk per category increase in smoking intensity or cumulative smoking.

1 Adjusted for sex and age at baseline.

2 Adjusted for sex, age at baseline, ancestry, past infectious mononucleosis, calendar year of diagnosis, disease phenotype, baseline EDSS, disease duration at baseline, and DMT exposure.

Bold values indicate statistical significance.

**Figure 1.**
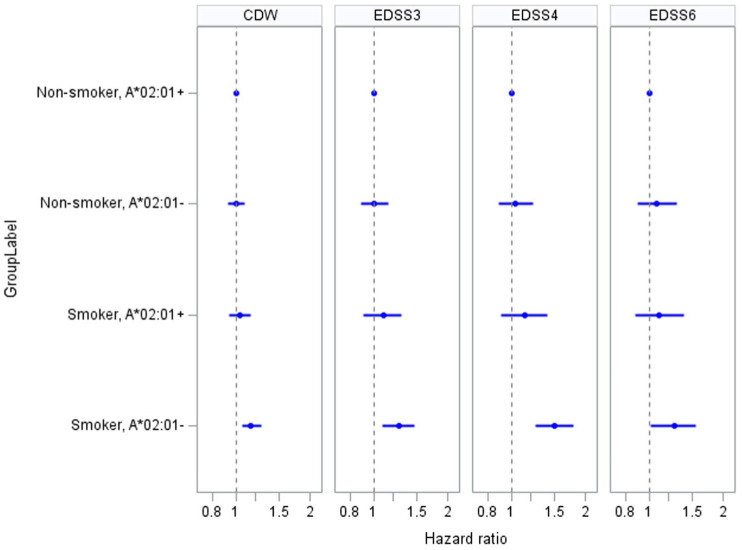
Hazard ratios for disability progression by HLA-A*02:01 and smoking status. HLA: human leukocyte antigen; EDSS: Expanded Disability Status Scale; CDW: confirmed disability worsening; DMT: disease-modifying therapy. Forest plot showing hazard ratios and 95% confidence intervals for CDW and EDSS 3, 4, and 6. The reference group is HLA-A*02:01-positive non-smokers. Adjusted for sex, ancestry, age at baseline, past infectious mononucleosis, calendar year of diagnosis, disease phenotype, baseline EDSS, disease duration at baseline, and proportion of follow-up spent on disease-modifying therapy.

**Figure 2.**
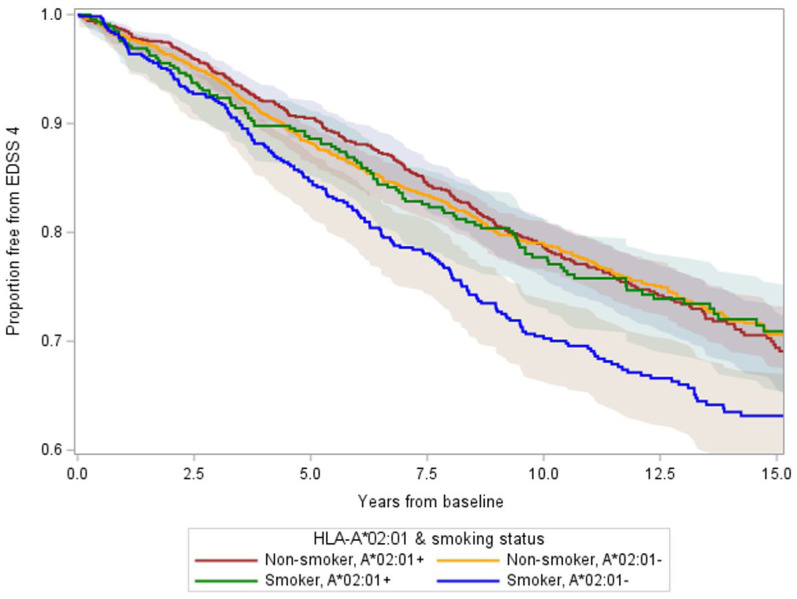
Time to EDSS 4, by HLA-A*02:01 and smoking status. EDSS: Expanded Disability Status Scale; HLA: human leukocyte antigen. Curves represent Kaplan–Meier survival estimates up to 15 years post-diagnosis. Groups are defined by smoking status at diagnosis (smokers vs non-smokers) and presence/absence of the HLA-A*02:01 allele. Shaded areas represent 95% confidence intervals. Numbers at risk at 0, 5, 10, and 15 years were as follows: non-smoker, A*02:01+: 1057, 870, 564, 231; non-smoker, A*02:01−: 1519, 1199, 845, 316; smoker, A*02:01+: 477, 370, 248, 118; smoker, A*02:01−: 636, 483, 323, 146.

When categorizing participants by smoking and HLA-A*02:01 status, using HLA-A*02:01-positive non-smokers as reference, smokers who lacked *HLA-A2:01* had an elevated risk of CDW (HR = 1.15, 95% CI = 1.06–1.26), while smoking had little impact within the HLA-A*02:01-positive group (HR = 1.04, 95% CI = 0.94–1.15). A similar pattern was observed for EDSS milestones. Smokers without HLA-A*02:01 had a higher risk of reaching EDSS 3 (HR = 1.26, 95% CI = 1.08–1.47) and EDSS 4 (HR = 1.50, 95% CI = 1.25–1.79), whereas smokers with HLA-A*02:01 showed no evidence of increased risk (Table 3, Figure 1). For EDSS 4, the AP indicated a significant departure from additivity (AP = 0.21, 95% CI = 0.01–0.42). Kaplan–Meier curves for time to EDSS 4 by smoking status and HLA-A*02:01 are shown in Figure 2. Analyses using EDSS 6 as an outcome showed a weaker overall association between smoking and disability progression, while the highest risk remained among individuals lacking HLA-A*02:01 (Table 3).

**Table 3.** HR with 95% CI of having unfavorable outcomes post-diagnosis, by HLA-A*02:01 status and smoking at diagnosis.

First clinical disease worsening (CDW)							
Smoker	A*02	*N*	Years (SD)	Outcome (%)	HR (95% CI)^a^	HR (95% CI)^b^	AP (95% CI)
−	+	1910	6.8 (5.0)	1191 (62.4)	1.0 (reference)	1.0 (reference)	
−	−	2601	6.8 (5.2)	1591 (61.2)	0.97 (0.85–1.10)	1.00 (0.93–1.08)	
+	+	1001	6.9 (5.4)	624 (62.3)	1.04 (0.87–1.23)	1.04 (0.94–1.15)	
+	−	1295	6.5 (5.3)	845 (65.3)	**1.19 (1.02–1.39)**	**1.15 (1.06–1.26)**	0.10 (−0.02; 0.21)
EDSS 3							
Smoker	A*02	*N*	Years (SD)	Outcome (%)	HR (95% CI)^a^	HR (95% CI)^b^	
−	+	1057	8.5 (5.3)	498 (47.1)	1.0 (reference)	1.0 (reference)	
−	−	1519	8.6 (5.6)	692 (45.6)	0.97 (0.87–1.09)	1.00 (0.89–1.15)	
+	+	477	8.5 (5.8)	223 (46.8)	1.02 (1.87–1.19)	1.09 (0.91–1.30)	
+	−	636	8.4 (5.8)	333 (52.4)	**1.15 (1.00–1.32)**	**1.26 (1.08–1.47)**	0.13 (−0.07; 0.33)
EDSS 4							
Smoker	A*02	*N*	Years (SD)	Outcome (%)	HR (95% CI)^a^	HR (95% CI)^b^	
−	+	1057	10.8 (5.2)	267 (25.3)	1.0 (reference)	1.0 (reference)	
−	−	1519	10.7 (5.4)	374 (24.6)	0.99 (0.84–1.16)	1.04 (0.89–1.22)	
+	+	477	10.9 (5.9)	125 (26.2)	1.03 (0.84–1.28)	1.13 (0.91–1.40)	
+	−	636	10.6 (5.7)	216 (34.0)	**1.37 (1.15–1.64)**	**1.50 (1.25–1.79)**	**0.21 (0.01; 0.42)**
EDSS 6							
Smoker	A*02	*N*	Years (SD)	Outcome (%)	HR (95% CI)^a^	HR (95% CI)^b^	
−	+	1587	6.5 (6.0)	189 (11.9)	1.0 (reference)	1.0 (reference)	
−	−	2185	6.5 (5.9)	257 (11.8)	1.03 (0.84–1.24)	1.07 (0.90–1.29)	
+	+	765	6.8 (6.0)	117 (15.3)	**1.35 (1.08–1.71)**	1.10 (0.88–1.39)	
+	−	1018	5.6 (5.4)	146 (14.3)	**1.53 (1.23–1.89)**	**1.26 (1.01–1.55)**	0.11 (−0.24; 0.27)

HLA: human leukocyte antigen; HR: hazard ratio; AP: attributable proportion due to interaction; CI: confidence interval; SD: standard deviation; CDW: clinical disease worsening; EDSS: Expanded Disability Status Scale.

a Adjusted for sex and age at baseline.

b Adjusted for sex, age at baseline, ancestry, past infectious mononucleosis, calendar year of diagnosis, disease phenotype, baseline EDSS, disease duration at baseline, and proportion of follow-up spent on disease-modifying therapy.

Bold values indicate statistical significance.

The joint effects of smoking at diagnosis and HLA-DRB1*15:01 status on disability progression are shown in Table 4. The risk of unfavorable outcomes was higher among smokers regardless of HLA-DRB1*15:01 status, although associations were weaker for EDSS 6. The AP was close to zero for all outcomes, indicating no evidence of interaction between smoking and HLA-DRB1*15:01 on an additive scale. Corresponding forest plots and Kaplan–Meier curves are shown in Supplemental eFigures 2 and 3.

**Table 4.** HR with 95% CI of having unfavorable outcomes post-diagnosis, by HLA-DRB1*15:01 status and smoking at diagnosis.

First clinical disease worsening (CDW)							
Smoker	DR*15	*N*	Years (SD)	Outcome (%)	HR (95% CI)^a^	HR (95% CI)^b^	AP (95% CI)
−	−	1928	6.8 (5.1)	1184 (61.4)	1.0 (reference)	1.0 (reference)	
−	+	2583	6.8 (5.1)	1598 (61.9)	1.01 (0.93–1.08)	1.01 (0.94–1.09)	
+	−	975	6.4 (5.1)	634 (65.0)	**1.13 (1.02–1.24)**	**1.15 (1.04–1.26)**	
+	+	1321	6.8 (5.4)	835 (63.2)	1.05 (0.96–1.14)	**1.09 (1.00–1.18)**	−0.09 (−0.24; 0.05)
EDSS 3							
Smoker	DR*15	*N*	Years (SD)	Outcome (%)	HR (95% CI)^a^	HR (95% CI)^b^	
−	−	1119	8.4 (5.4)	506 (45.2)	1.0 (reference)	1.0 (reference)	
−	+	1457	8.6 (5.6)	684 (47.0)	1.07 (0.94–1.22)	1.07 (0.94–1.22)	
+	−	489	8.4 (5.9)	233 (47.7)	1.13 (0.95–1.34)	**1.18 (1.00–1.40)**	
+	+	624	8.4 (5.7)	323 (51.8)	**1.24 (1.06–1.45)**	**1.27 (1.09–1.49)**	0.01 (−0.25; 0.28)
EDSS 4							
Smoker	DR*15	*N*	Years (SD)	Outcome (%)	HR (95% CI)^a^	HR (95% CI)^b^	
−	−	1119	10.5 (5.2)	269 (24.0)	1.0 (reference)	1.0 (reference)	
−	+	1457	10.9 (5.4)	372 (25.5)	1.02 (0.87–1.19)	1.05 (0.90–1.23)	
+	−	489	10.6 (5.9)	149 (30.5)	**1.24 (1.01–1.51)**	**1.34 (1.10–1.64)**	
+	+	624	10.8 (5.7)	192 (30.8)	**1.26 (1.05–1.51)**	**1.35 (1.12–1.62)**	−0.04 (−0.29; 0.21)
EDSS 6							
Smoker	DR*15	*N*	Years (SD)	Outcome (%)	HR (95% CI)^a^	HR (95% CI)^b^	
−	−	1632	6.6 (5.9)	212 (13.0)	1.0 (reference)	1.0 (reference)	
−	+	2140	6.5 (6.0)	234 (10.9)	0.88 (0.72–1.05)	0.90 (0.74–1.06)	
+	−	770	6.0 (5.7)	117 (15.2)	**1.40 (1.11–1.75)**	1.19 (0.93–1.45)	
+	+	1013	6.3 (5.9)	146 (14.4)	**1.28 (1.04–1.58)**	1.11 (0.90–1.32)	0.02 (−0.35; 0.26)

HLA: human leukocyte antigen; HR: hazard ratio; AP: attributable proportion due to interaction; CI: confidence interval; SD: standard deviation; CDW: clinical disease worsening; EDSS: Expanded Disability Status Scale.

a Adjusted for sex and age at baseline.

b Adjusted for sex, age at baseline, ancestry, past infectious mononucleosis, calendar year of diagnosis, disease phenotype, baseline EDSS, disease duration at baseline, and proportion of follow-up spent on disease-modifying therapy.

Bold values indicate statistical significance.

### Sensitivity analyses

Analyses limited to participants of Nordic ancestry yielded results consistent with the main findings. The increased risk associated with smoking was most pronounced among HLA-A*02:01-negative individuals, with no meaningful differences among HLA-A*02:01-positive smokers (Supplemental eTable 4). Smoking increased the risk of disability outcomes regardless of HLA-DRB1*15:01 status, with no evidence of interaction (Supplemental eTable 5).

Findings also remained consistent when the analyses were limited to DMT-treated participants (Supplemental eTables 6 and 7) and to incident cases with further adjustment for demographic and lifestyle covariates (Supplemental eTables 8 and 9). Estimates remained unchanged after further exclusion of genetic outliers and additional adjustment for principal component vectors (data not shown).

## Discussion

In this large, population-based cohort of individuals with MS, we found that smoking at diagnosis was associated with an increased risk of objectively measured disability progression primarily among those who lacked the HLA-A*02:01 allele. This pattern was observed consistently across CDW and EDSS milestones, whereas no evidence of effect modification was observed for HLA-DRB1*15:01. These findings suggest that genetic susceptibility to the harmful effects of smoking on MS progression may be restricted to specific HLA class I alleles.

Smoking is one of the most consistently identified environmental determinants of both MS onset and progression.^4–7^ Proposed mechanisms include persistent systemic inflammation, oxidative stress, impaired mitochondrial function, and enhanced microglial activation,^4^ all of which are closely linked to neurodegeneration. Smoking also induces chronic airway irritation and peripheral immune activation, which may shape adaptive immune responses.^19^ Together, these mechanisms align with current models that integrate innate immune activation within the central nervous system with peripheral immune contributions in driving disability accumulation.^20^ Yet the extent to which these smoking-related pathways interact with genetically defined immune responses has remained unclear. Although smoking-related immune alterations may persist after cessation,^21^ several studies suggest that the detrimental effect of smoking on MS progression is strongest for ongoing smoking,^7,13^ and that smoking cessation is associated with a more favorable prognosis.^13^ This supports our focus on current smoking at diagnosis as the most relevant exposure for disability progression.

Although the HLA region is central to MS susceptibility,^14^ previous genome-wide studies show limited evidence that common HLA alleles, including HLA-DRB1*15:01, substantially influence post-onset disease course.^8^ Our findings align with this view for HLA-DRB1*15:01. In contrast, the clear pattern observed for HLA-A*02:01 suggests that class I-restricted immune pathways may modulate how environmental exposures influence neuroinflammatory activity. HLA-A*02:01 is associated with reduced MS risk,^14^ with proposed mechanisms involving antigen processing and cytotoxic T-cell responses to Epstein–Barr virus (EBV). Recent data also indicate that class I alleles exert broader immunoregulatory effects, including altered type I interferon signaling.^22^ The absence of HLA-A*02:01 might, therefore, reflect a genetically defined vulnerability to environmental insults that amplify chronic inflammatory damage. For EDSS 6, the overall effect of smoking on disability progression was weaker, with the highest risk observed among individuals lacking HLA-A*02:01. This is consistent with later stages of disability accumulation being increasingly influenced by neurodegenerative and compartmentalized inflammatory processes,^23,24^ which may be less responsive to external influences. These findings support a model in which environmental exposures exert their effects within genetically shaped immune-regulatory pathways, rather than acting uniformly across the MS population. In this context, smoking-induced immune activation may have a greater impact on HLA-A*02:01-negative individuals. This provides a rationale for further mechanistic studies into gene–environment interplay in neuroinflammation and neurodegeneration.

Standard genome-wide association studies (GWAS) primarily estimate marginal genetic effects and do not explicitly model gene–environment interactions. Variants whose impact is mainly expressed through interaction with environmental exposures may, therefore, show weak or no marginal associations and be difficult to detect in conventional GWAS.^25,26^ In our cohort, the interaction was driven by a specific subgroup of patients (HLA-A*02:01-negative smokers), and such subgroup-specific effects are easily attenuated when averaged across heterogeneous cohorts in large international GWAS. Differences in the population frequency of A*02:01 across countries may further dilute subgroup effects.^27^ Thus, the absence of a clear HLA signal in severity GWAS does not necessarily contradict the presence of an interaction between smoking and HLA-A*02:01.

Some limitations should be acknowledged. Smoking status at diagnosis was self-reported but is expected to be subject to limited recall bias in EIMS, whereas information in GEMS was collected retrospectively. Although this increases the potential for recall error in GEMS, the consistency of results when restricting analyses to incident cases argues that substantial differential misclassification is unlikely. We did not model changes in smoking behavior during follow-up; however, we have previously observed that relatively few individuals discontinued smoking after diagnosis, suggesting that post-diagnosis changes in smoking habits are unlikely to have influenced the observed associations in any substantial way.

HLA genotyping was available for 79% of the participants, raising the possibility of selection bias. However, smoking prevalence at diagnosis did not differ between individuals with and without HLA data, suggesting that missing genotypes are not likely to have introduced major bias in the smoking-genotype analyses.

Residual confounding remains possible despite adjustment for a wide range of clinical and lifestyle factors, although the consistency of findings across multiple adjusted models argues against major bias. HLA alleles beyond HLA-A*02:01 and HLA-DRB1*15:01 were not evaluated, and we can, therefore, not rule out contributions from other alleles that might influence susceptibility to smoking-related neuroinflammatory mechanisms.

Finally, mechanistic interpretations remain hypothetical and require validation in immunological, virological, or experimental models. Although the interaction between smoking and HLA-A*02:01 was consistent across outcomes, the possibility of a chance finding remains, and confirmation in independent cohorts is warranted. In conclusion, our findings indicate that the deleterious effects of smoking on MS disability progression are modified by HLA-A*02:01 status, suggesting that gene–environmental interactions relevant for MS susceptibility may also extend to disease course. The findings underscore the importance of avoiding smoking in individuals with MS, particularly among those lacking HLA-A*02:01, and motivate further mechanistic studies into class I-mediated pathways in neuroinflammation and neurodegeneration.

## Supplemental Material

sj-docx-1-msj-10.1177_13524585261453243 – Supplemental material for Interaction between smoking and HLA-A*02:01 in multiple sclerosis progression Supplemental material, sj-docx-1-msj-10.1177_13524585261453243 for Interaction between smoking and HLA-A*02:01 in multiple sclerosis progression by Anna Karin Hedström, Pernilla Stridh, Jan Hillert, Tomas Olsson and Lars Alfredsson in Multiple Sclerosis Journal
