# Supplementary material for: Interaction between smoking and HLA-A*02:01 in multiple sclerosis progression
Source: Mult Scler. 2026 Jul 3;32(9):962–71. doi: 10.1177/13524585261453243 (PMC13385633; doi:10.1177/13524585261453243)
Supplement: sj-docx-1-msj-10.1177_13524585261453243 – Supplemental material for Interaction between smoking and HLA-A*02:01 in multiple sclerosis progression [file sj-docx-1-msj-10.1177_13524585261453243.docx]

**Supplementary files**

eTable 1. Median follow-up time (years) across combined smoking and HLA strata.

eTable 2. Characteristics of overall sample and by HLA-A*02:01 status.

eTable 3. Characteristics of overall sample and by HLA-DRB1*15:01 status.

eTable 4. Sensitivity analysis: HR with 95% CI of having unfavorable outcomes post-diagnosis, by HLA-A*02:01 status and smoking at diagnosis, limited to cases of Nordic origin.

eTable 5. Sensitivity analysis: HR with 95% CI of having unfavorable outcomes post-diagnosis, by HLA-DRB1*15:01 status and smoking at diagnosis, limited to cases of Nordic origin.

eTable 6. Sensitivity analysis: HR with 95% CI of having unfavorable outcomes post-diagnosis, by HLA-A*02:01 status and smoking at diagnosis, limited to DMT-treated cases.

eTable 7. Sensitivity analysis: HR with 95% CI of having unfavorable outcomes post-diagnosis, by HLA-DRB1*15:01 status and smoking at diagnosis, limited to DMT-treated cases.

eTable 8. Sensitivity analysis: HR with 95% CI of having unfavorable outcomes post-diagnosis, by HLA-A*02:01 status and smoking at diagnosis, limited to incident cases.

eTable 9. Sensitivity analysis: HR with 95% CI of having unfavorable outcomes post-diagnosis, by HLA-DRB1*15:01 status and smoking at diagnosis, limited to incident cases.

eFigure 1. Flow chart

eFigure 2. Hazard ratios for disability progression by HLA-DRB1*15:01 and smoking status.

eFigure 3. Time to EDSS 4, by HLA-DRB1*15:01 and smoking status

eTable 1. Median follow-up time (years) across combined smoking and HLA strata.

| HLA-A*02:01 | HLA-DRB1*15:01 | Median follow-up time (range) |
| --- | --- | --- |
| - | - | 11.3 (6.6-15.4) |
| + | - | 11.2 (6.2-15.2) |
| - | + | 10.9 (6.0-15.1) |
| + | + | 11.0 (6.3-15.4) |
| HLA-A*02:01 | Smoking |  |
| - | - | 11.0 (6.2-15.2) |
| + | - | 10.8 (6.2-15.1) |
| - | + | 11.2 (6.2-15.3) |
| + | + | 11.4 (6.4-15.6) |
| HLA-DRB1*15:01 | Smoking |  |
| - | - | 11.1 (6.5-15.2) |
| + | - | 10.7 (6.1-15.2) |
| - | + | 11.6 (6.5-15.5) |
| + | + | 11.3 (6.3-15.5) |

HLA=human leukocyte antigen

eTable 2. Characteristics of overall sample and by HLA-A*02:01 status.

|  | | | Total | HLA-A*02:01+ | HLA-A*02:01- | P value |
| --- | --- | --- | --- | --- | --- | --- |
| N | | | 6807 | 2911 | 3896 |  |
| Median follow-up (range) | | | 11.1 (7.2-16.3) | 11.1 (6.3-15.4) | 11.0 (6.2-15.3) |  |
| Age at disease onset (SD) | | | 33.3 (10.6) | 33.7 (10.6) | 33.1 (10.6) | 0.03 |
| Age at diagnosis (SD) | | | 37.8 (11.3) | 38.0 (11.5) | 37.6 (11.2) | 0.14 |
| Sex, n (%) | Female | | 4956 (72.8) | 2110 (72.5) | 2846 (73.0) | 0.60 |
|  | Male | | 1851 (27.2) | 801 (27.5) | 1050 (27.0) |  |
| Ancestry, n (%) | Nordic | | 5967 (87.7) | 2625 (90.2) | 3342 (85.8) | <0.0001 |
|  | Non-Nordic | | 840 (12.3) | 286 (9.8) | 554 (14.2) |  |
| MS type, n (%) | Relapsing-onset | | 6159 (90.5) | 2608 (89.6) | 3551 (91.1) | 0.50 |
|  | Progressive-onset | | 569 (8.4) | 260 (8.9) | 309 (7.9) |  |
|  | Unknown | | 79 (1.2) | 43 (1.5) | 36 (0.9) |  |
| DMT, n (%) | Never | | 1646 (24.2) | 712 (24.5) | 934 (24.0) | 0.52 |
|  | Platform | | 2831 (41.6) | 1216 (41.8) | 1615 (41.5) |  |
|  | High-efficacy | | 1663 (24.4) | 285 (9.8) | 965 (9.8) |  |
|  | Escalation | | 667 (9.8) | 698 (24.0) | 382 (24.8) |  |
| Time to DMT initiation, years (SD) | | | 2.2 (5.2) | 2.3 (5.2) | 2.1 (5.2) | 0.29 |
| Baseline EDSS (SD) | | | 2.7 (2.0) | 2.8 (2.0) | 2.7 (2.0) | 0.004 |
| Baseline MSIS-PHYS (SD) | | | 26.8 (23.5) | 26.5 (23.3) | 27.0 (23.7) | 0.50 |
| Smoking at diagnosis, n (%) | | Yes | 2296 (33.7) | 1001 (34.4) | 1295 (33.2) | 0.32 |
|  |  | No | 4511 (66.3) | 1910 (65.6) | 2601 (66.8) |  |
| Pack years of smoking (SD) | | | 9.6 (10.1) | 9.3 (9.3) | 9.7 (10.6) | 0.40 |
| Past IM, n (%) | | Yes | 686 (13.8) | 323 (11.1) | 590 (15.1) | 0.0009 |
|  |  | No | 3490 (70.4) | 2118 (72.8) | 2663 (68.4) |  |
|  |  | Unsure | 780 (15.7) | 470 (16.2) | 643 (16.5) |  |
| Body mass index, kg/m^2^ (SD) | | | 24.9 (4.5) | 25.0 (4.7) | 24.8 (4.4) | 0.63 |
| HLA-DRB*15:01 status, n (%) | | Positive | 3904 (57.4) | 1755 (60.3) | 2149 (55.2) | <0.0001 |
|  |  | Negative | 2903 (42.7) | 1156 (39.7) | 1747 (44.8) |  |

DMT=disease-modifying therapy; EDSS=Expanded Disability Status Scale; SD=standard deviation; MSIS-PHYS=Multiple Sclerosis Impact Scale physical component; IM=infectious mononucleosis; HLA=human leuokocyte antigen.

eTable 3. Characteristics of overall sample and by HLA-DRB1*15:01 status.

|  | | | Total | DR15*15:01+ | DRB1*15:01- | P value |
| --- | --- | --- | --- | --- | --- | --- |
| N | | | 6807 | 3904 | 2903 |  |
| Median follow-up (range) | | | 11.1 (7.2-16.3) | 10.9 (6.2-15.3) | 11.2 (6.5-15.3) |  |
| Age at disease onset (SD) | | | 33.3 (10.6) | 32.9 (10.5) | 33.9 (10.6) | 0.003 |
| Age at diagnosis (SD) | | | 37.8 (11.3) | 37.4 (11.2) | 38.2 (11.4) | 0.004 |
| Sex, n (%) | Female | | 4956 (72.8) | 2920 (74.8) | 2036 (70.1) | <0.0001 |
|  | Male | | 1851 (27.2) | 984 (25.2) | 867 (29.9) |  |
| Ancestry, n (%) | Nordic | | 5967 (87.7) | 3552 (91.0) | 2415 (83.2) | <0.0001 |
|  | Non-Nordic | | 840 (12.3) | 352 (9.0) | 488 (16.8) |  |
| MS type, n (%) | Relapsing-onset | | 6159 (90.5) | 3553 (91.0) | 2606 (89.8) | 0.59 |
|  | Progressive-onset | | 569 (8.4) | 313 (8.0) | 256 (8.8) |  |
|  | Unknown | | 79 (1.2) | 38 (1.0) | 41 (1.4) |  |
| DMT, n (%) | Never | | 1646 (24.2) | 959 (24.6) | 687 (23.7) | 0.16 |
|  | Platform | | 2831 (41.6) | 1633 (41.8) | 1198 (41.3) |  |
|  | High-efficacy | | 1663 (24.4) | 366 (9.4) | 301 (10.4) |  |
|  | Escalation | | 667 (9.8) | 946 (24.2) | 717 (24.7) |  |
| Time to DMT initiation, years (SD) | | | 2.2 (5.2) | 2.2 (5.3) | 2.1 (5.1) | 0.28 |
| Baseline EDSS (SD) | | | 2.7 (2.0) | 2.8 (2.0) | 2.7 (2.0) | 0.04 |
| Baseline MSIS-PHYS (SD) | | | 26.8 (23.5) | 26.8 (23.4) | 26.7 (23.7) | 0.87 |
| Smoking at diagnosis, n (%) | | Yes | 2296 (33.7) | 1321 (33.8) | 975 (33.6) | 0.82 |
|  |  | No | 4511 (66.3) | 2583 (66.2) | 1928 (66.4) |  |
| Pack years of smoking (SD) | | | 9.6 (10.1) | 9.2 (10.4) | 10.0 (9.6) | 0.01 |
| Past IM, n (%) | | Yes | 686 (13.8) | 520 (13.3) | 393 (13.5) | 0.31 |
|  |  | No | 3490 (70.4) | 2760 (70.7) | 2021 (69.6) |  |
|  |  | Unsure | 780 (15.7) | 624 (16.0) | 489 (16.8) |  |
| Body mass index, kg/m^2^ (SD) | | | 24.9 (4.5) | 24.9 (4.6) | 24.9 (4.5) | 0.98 |
| HLA-A*02:01 status, n (%) | | Positive | 2911 (42.3) | 1755 (45.0) | 1156 (39.8) | 0.0003 |
|  |  | Negative | 3896 (57.2) | 2149 (55.0) | 1747 (60.2) |  |

DMT=disease-modifying therapy; EDSS=Expanded Disability Status Scale; SD=standard deviation; MSIS-PHYS=Multiple Sclerosis Impact Scale physical component; IM=infectious mononucleosis; HLA=human leuokocyte antigen.

eTable 4. Sensitivity analysis: HR with 95% CI of having unfavorable outcomes post-diagnosis, by HLA-A*02:01 status and smoking at diagnosis, limited to cases of Nordic origin.

| First clinical disease worsening (CDW) | | | | | | |  |
| --- | --- | --- | --- | --- | --- | --- | --- |
| Smoker | A*02:01 | N | Years (SD) | Outcome (%) | HR (95% CI)^1^ | HR (95% CI)^2^ | AP (95% CI) |
| - | + | 1737 | 6.8 (5.0) | 1083 (62.4) | 1.0 (reference) | 1.0 (reference) |  |
| - | - | 2228 | 6.9 (5.2) | 1346 (60.4) | 0.97 (0.90-1.05) | 0.97 (0.90-1.05) |  |
| + | + | 888 | 7.0 (5.4) | 546 (61.5) | 1.00 (0.90-1.11) | 1.00 (0.90-1.11) |  |
| + | - | 1114 | 6.4 (5.2) | 719 (64.5) | **1.12 (1.02-1.23)** | **1.12 (1.02-1.23)** | 0.12 (-0.01; 0.28) |
| EDSS 3 | | | | | | |  |
| Smoker | A*02:01 | N | Years (SD) | Outcome (%) | HR (95% CI)^1^ | HR (95% CI)^2^ |  |
| - | + | 943 | 8.5 (5.3) | 448 (47.5) | 1.0 (reference) | 1.0 (reference) |  |
| - | - | 1282 | 8.6 (5.6) | 579 (45.2) | 0.95 (0.83-1.10) | 0.97 (0.84-1.10) |  |
| + | + | 422 | 8.6 (5.8) | 199 (47.2) | 1.06 (0.88-1.28) | 1.07 (0.89-1.29) |  |
| + | - | 524 | 8.3 (5.8) | 283 (54.0) | **1.29 (1.09-1.52)** | **1.31 (1.11-1.55)** | **0.18 (0.01; 0.40)** |
| EDSS 4 | | | | | | |  |
| Smoker | A*02:01 | N | Years (SD) | Outcome (%) | HR (95% CI)^1^ | HR (95% CI)^2^ |  |
| - | + | 943 | 10.8 (5.3) | 248 (26.3) | 1.0 (reference) | 1.0 (reference) |  |
| - | - | 12182 | 10.7 (5.5) | 308 (24.0) | 0.94 (0.79-1.11) | 0.97 (0.82-1.15) |  |
| + | + | 422 | 11.0 (5.9) | 113 (26.8) | 1.07 (0.86-1.34) | 1.11 (0.88-1.38) |  |
| + | - | 524 | 10.5 (5.7) | 184 (35.1) | **1.47 (1.21-1.78)** | **1.54 (1.27-1.87)** | **0.28 (0.08; 0.48)** |

HLA=human leukocyte antigen; HR=hazard ratio; AP=attributable proportion due to interaction; CI=confidence interval; SD=standard deviation, CDW=clinical disease worsening; EDSS=expanded disability status scale. ^1^adjusted for sex and age at baseline; ^2^adjusted for sex, age at baseline, past infectious mononucleosis; calendar year of diagnosis, disease phenotype; baseline EDSS, disease duration at baseline, and proportion of follow-up spent on disease-modifying therapy.

eTable 5. Sensitivity analysis: HR with 95% CI of having unfavorable outcomes post-diagnosis, by HLA-DRB1*15:01 status and smoking at diagnosis, limited to cases of Nordic origin.

| First clinical disease worsening (CDW) | | | | | | |  |
| --- | --- | --- | --- | --- | --- | --- | --- |
| Smoker | DRB1*15:01 | N | Years (SD) | Outcome (%) | HR (95% CI)^1^ | HR (95% CI)^2^ | AP (95% CI) |
| - | - | 1616 | 6.8 (5.1) | 988 (61.1) | 1.0 (reference) | 1.0 (reference) |  |
| - | + | 2349 | 6.8 (5.1) | 1441 (61.4) | 1.01 (0.93-1.10) | 1.00 (0.93-1.09) |  |
| + | - | 799 | 6.6 (5.1) | 509 (63.7) | 1.11 (1.00-1.24) | 1.11 (0.99-1.23) |  |
| + | + | 1203 | 6.8 (5.5) | 756 (62.8) | 1.09 (0.98-1.18) | 1.10 (0.98-1.18) | -0.03 (-0.17; 0.11) |
| EDSS 3 | | | | | | |  |
| Smoker | DRB1*15:01 | N | Years (SD) | Outcome (%) | HR (95% CI)^1^ | HR (95% CI)^2^ |  |
| - | - | 912 | 8.5 (5.4) | 415 (45.5) | 1.0 (reference) | 1.0 (reference) |  |
| - | + | 1313 | 8.6 (5.5) | 612 (46.6) | 1.09 (0.95-1.26) | 1.07 (0.93-1.23) |  |
| + | - | 386 | 8.4 (5.8) | 187 (48.5) | **1.20 (1.00-1.45)** | **1.23 (1.02-1.50)** |  |
| + | + | 560 | 8.4 (5.8) | 295 (52.7) | **1.35 (1.14-1.60)** | **1.31 (1.11-1.55)** | 0.01 (-0.14; 0.15) |
| EDSS 4 | | | | | | |  |
| Smoker | DRB1*15:01 | N | Years (SD) | Outcome (%) | HR (95% CI)^1^ | HR (95% CI)^2^ |  |
| - | - | 912 | 10.6 (5.3) | 225 (24.7) | 1.0 (reference) | 1.0 (reference) |  |
| - | + | 1313 | 10.9 (5.4) | 331 (25.2) | 1.03 (0.87-1.22) | 1.02 (0.86-1.21) |  |
| + | - | 386 | 10.6 (6.0) | 121 (31.4) | **1.31 (1.05-1.64)** | **1.39 (1.11-1.73)** |  |
| + | + | 560 | 10.8 (5.7) | 176 (31.4) | **1.39 (1.14-1.70)** | **1.38 (1.13-1.69)** | -0.02 (-0.28; 0.25) |

HLA=human leukocyte antigen; HR=hazard ratio; AP=attributable proportion due to interaction; CI=confidence interval; SD=standard deviation, CDW=clinical disease worsening; EDSS=expanded disability status scale. ^1^adjusted for sex and age at baseline; ^2^adjusted for sex, age at baseline, past infectious mononucleosis; calendar year of diagnosis, disease phenotype; baseline EDSS, disease duration at baseline, and proportion of follow-up spent on disease-modifying therapy.

eTable 6. Sensitivity analysis: HR with 95% CI of having unfavorable outcomes post-diagnosis, by HLA-A*02:01 status and smoking at diagnosis, limited to DMT-treated cases.

| First clinical disease worsening (CDW) | | | | | | |  |
| --- | --- | --- | --- | --- | --- | --- | --- |
| Smoker | A*02:01 | N | Years (SD) | Outcome (%) | HR (95% CI)^1^ | HR (95% CI)^2^ | AP (95% CI) |
| - | + | 1549 | 6.9 (5.1) | 983 (63.5) | 1.0 (reference) | 1.0 (reference) |  |
| - | - | 2137 | 6.9 (5.2) | 1354 (63.4) | 1.04 (0.94-1.16) | 1.04 (0.93-1.15) |  |
| + | + | 769 | 6.8 (5.4) | 505 (65.7) | 1.11 (0.98-1.25) | 1.11 (0.98-1.25) |  |
| + | - | 1022 | 6.3 (5.2) | 705 (69.0) | **1.23 (1.10-1.38)** | **1.22 (1.09-1.37)** | 0.07 (-0.09; 0.26) |
| EDSS 3 | | | | | | |  |
| Smoker | A*02:01 | N | Years (SD) | Outcome (%) | HR (95% CI)^1^ | HR (95% CI)^2^ |  |
| - | + | 951 | 8.7 (5.3) | 446 (46.9) | 1.0 (reference) | 1.0 (reference) |  |
| - | - | 1365 | 8.6 (5.6) | 623 (45.6) | 1.00 (0.87-1.14) | 1.01 (0.88-1.15) |  |
| + | + | 415 | 8.3 (5.8) | 200 (48.2) | 1.16 (0.96-1.39) | 1.16 (0.96-1.39) |  |
| + | - | 561 | 8.3 (5.8) | 309 (55.1) | **1.33 (1.13-1.56)** | **1.33 (1.13-1.56)** | 0.12 (-0.08; 0.32) |
| EDSS 4 | | | | | | |  |
| Smoker | A*02:01 | N | Years (SD) | Outcome (%) | HR (95% CI)^1^ | HR (95% CI)^2^ |  |
| - | + | 951 | 10.9 (5.1) | 242 (25.5) | 1.0 (reference) | 1.0 (reference) |  |
| - | - | 1365 | 10.8 (5.3) | 334 (24.5) | 1.00 (0.85-1.18) | 1.02 (0.86-1.20) |  |
| + | + | 415 | 10.9 (5.9) | 108 (26.0) | 1.08 (0.86-1.36) | 1.09 (0.87-1.37) |  |
| + | - | 561 | 10.7 (5.6) | 199 (35.5) | **1.52 (1.26-1.84)** | **1.53 (1.27-1.85)** | **0.28 (0.08; 0.48)** |

HLA=human leukocyte antigen; HR=hazard ratio; AP=attributable proportion due to interaction; CI=confidence interval; SD=standard deviation, CDW=clinical disease worsening; EDSS=expanded disability status scale. ^1^adjusted for sex and age at baseline; ^2^adjusted for sex, age at baseline, ancestry; past infectious mononucleosis; calendar year of diagnosis, disease phenotype; baseline EDSS, disease duration at baseline, and DMT exposure as a time-updated variable.

eTable 7. Sensitivity analysis: HR with 95% CI of having unfavorable outcomes post-diagnosis, by HLA-DRB1*15:01 status and smoking at diagnosis, limited to DMT-treated cases.

| First clinical disease worsening (CDW) | | | | | | |  |
| --- | --- | --- | --- | --- | --- | --- | --- |
| Smoker | DRB1*15:01 | N | Years (SD) | Outcome (%) | HR (95% CI)^1^ | HR (95% CI)^2^ | AP (95% CI) |
| - | - | 1581 | 6.8 (5.1) | 1007 (63.7) | 1.0 (reference) | 1.0 (reference) |  |
| - | + | 2105 | 6.9 (5.2) | 1330 (63.2) | 0.99 (0.91-1.08) | 0.99 (0.92-1.08) |  |
| + | - | 778 | 6.3 (5.1) | 540 (69.4) | **1.20 (1.08-1.33)** | **1.20 (1.07-1.32)** |  |
| + | + | 1013 | 6.7 (5.4) | 670 (66.1) | **1.10 (1.00-1.22)** | **1.11 (1.00-1.21)** | -0.08 (-0.23; 0.06) |
| EDSS 3 | | | | | | |  |
| Smoker | DRB1*15:01 | N | Years (SD) | Outcome (%) | HR (95% CI)^1^ | HR (95% CI)^2^ |  |
| - | - | 997 | 8.5 (5.4) | 454 (45.5) | 1.0 (reference) | 1.0 (reference) |  |
| - | + | 1319 | 8.8 (5.6) | 615 (46.6) | 1.01 (0.90-1.14) | 1.00 (0.88-1.13) |  |
| + | - | 437 | 8.4 (5.9) | 217 (49.7) | 1.14 (0.96-1.33) | 1.16 (0.98-1.35) |  |
| + | + | 539 | 8.2 (5.7) | 292 (54.2) | **1.32 (1.14-1.53)** | **1.25 (1.08-1.45)** | 0.08 (0.10; 0.27) |
| EDSS 4 | | | | | | |  |
| Smoker | DRB1*15:01 | N | Years (SD) | Outcome (%) | HR (95% CI)^1^ | HR (95% CI)^2^ |  |
| - | - | 997 | 10.6 (5.1) | 243 (24.4) | 1.0 (reference) | 1.0 (reference) |  |
| - | + | 1319 | 11.1 (5.4) | 333 (25.3) | 1.00 (0.85-1.18) | 0.99 (0.84-1.17) |  |
| + | - | 437 | 10.7 (5.9) | 137 (31.4) | **1.29 (1.04-1.59)** | **1.31 (1.06-1.62)** |  |
| + | + | 539 | 10.8 (5.6) | 170 (31.5) | **1.38 (1.13-1.68)** | **1.32 (1.08-1.61)** | 0.01 (-0.25; 0.27) |

HLA=human leukocyte antigen; HR=hazard ratio; AP=attributable proportion due to interaction; CI=confidence interval; SD=standard deviation, CDW=clinical disease worsening; EDSS=expanded disability status scale. ^1^adjusted for sex and age at baseline; ^2^adjusted for sex, age at baseline, ancestry; past infectious mononucleosis; calendar year of diagnosis, disease phenotype; baseline EDSS, disease duration at baseline, and DMT exposure as a time-updated variable.

eTable 8. Sensitivity analysis: HR with 95% CI of having unfavorable outcomes post-diagnosis, by HLA-A*02:01 status and smoking at diagnosis, limited to incident cases.

| First clinical disease worsening (CDW) | | | | | | |  |
| --- | --- | --- | --- | --- | --- | --- | --- |
| Smoker | A*02:01 | N | Years (SD) | Outcome (%) | HR (95% CI)^1^ | HR (95% CI)^2^ | AP (95% CI) |
| - | + | 773 | 6.6 (4.5) | 409 (52.9) | 1.0 (reference) | 1.0 (reference) |  |
| - | - | 1111 | 6.9 (4.7) | 589 (53.0) | 0.95 (0.84-1.08) | 0.97 (0.85-1.09) |  |
| + | + | 262 | 6.4 (4.6) | 142 (54.2) | 1.03 (0.85-1.25) | 1.04 (0.86-1.26) |  |
| + | - | 329 | 6.1 (4.4) | 192 (58.4) | **1.21 (1.02-1.44)** | **1.23 (1.03-1.46)** | 0.18 (-0.04; 0.38) |
| EDSS 3 | | | | | | |  |
| Smoker | A*02:01 | N | Years (SD) | Outcome (%) | HR (95% CI)^1^ | HR (95% CI)^2^ |  |
| - | + | 559 | 8.2 (4.6) | 196 (35.1) | 1.0 (reference) | 1.0 (reference) |  |
| - | - | 803 | 8.3 (4.8) | 284 (35.4) | 0.99 (0.81-1.21) | 0.99 (0.81-1.21) |  |
| + | + | 200 | 7.7 (4.9) | 66 (33.0) | 1.00 (0.73-1.37) | 0.99 (0.72-1.35) |  |
| + | - | 229 | 7.9 (4.6) | 95 (41.5) | **1.31 (1.00-1.72)** | **1.32 (1.00-1.74)** | 0.26 (-0.05; 0.56) |
| EDSS 4 | | | | | | |  |
| Smoker | A*02:01 | N | Years (SD) | Outcome (%) | HR (95% CI)^1^ | HR (95% CI)^2^ |  |
| - | + | 559 | 9.6 (4.2) | 92 (16.5) | 1.0 (reference) | 1.0 (reference) |  |
| - | - | 803 | 9.9 (4.4) | 134 (16.7) | 0.98 (0.75-1.27) | 0.98 (0.75-1.28) |  |
| + | + | 200 | 9.5 (4.4) | 30 (15.0) | 0.95 (0.63-1.44) | 0.95 (0.63-1.43) |  |
| + | - | 229 | 9.5 (4.2) | 52 (22.7) | **1.53 (1.08-2.15)** | **1.51 (1.07-2.14)** | **0.38 (0.01; 0.69)** |

HLA=human leukocyte antigen; HR=hazard ratio; AP=attributable proportion due to interaction; CI=confidence interval; SD=standard deviation, CDW=clinical disease worsening; EDSS=expanded disability status scale. ^1^adjusted for sex and age at baseline; ^2^adjusted for sex, age at baseline, ancestry; past infectious mononucleosis; calendar year of diagnosis, disease phenotype; baseline EDSS, disease duration at baseline, proportion of follow-up spent on disease-modifying therapy; educational attainment; body mass index; alcohol consumption; fish consumption; sun exposure habits; and physical activity.

eTable 9. Sensitivity analysis: HR with 95% CI of having unfavorable outcomes post-diagnosis, by HLA-DRB1*15:01 status and smoking at diagnosis, limited to incident cases.

| First clinical disease worsening (CDW) | | | | | | |  |
| --- | --- | --- | --- | --- | --- | --- | --- |
| Smoker | DRB1*15:01 | N | Years (SD) | Outcome (%) | HR (95% CI)^1^ | HR (95% CI)^2^ | AP (95% CI) |
| - | + | 862 | 6.8 (4.6) | 448 (52.0) | 1.0 (reference) | 1.0 (reference) |  |
| - | - | 1022 | 6.8 (4.6) | 550 (53.8) | 1.04 (0.93-1.18) | 1.03 (0.92-1.16) |  |
| + | + | 268 | 5.9 (4.4) | 157 (58.6) | **1.22 (1.03-1.44)** | **1.20 (1.01-1.42)** |  |
| + | - | 323 | 6.5 (4.6) | 177 (54.8) | 1.15 (0.96-1.32) | 1.16 (0.97-1.33) | -0.08 (-0.36; 0.17) |
| EDSS 3 | | | | | | |  |
| Smoker | DRB1*15:01 | N | Years (SD) | Outcome (%) | HR (95% CI)^1^ | HR (95% CI)^2^ |  |
| - | + | 614 | 8.2 (4.7) | 207 (33.7) | 1.0 (reference) | 1.0 (reference) |  |
| - | - | 748 | 8.2 (4.8) | 273 (36.5) | 1.10 (0.88-1.25) | 1.09 (0.86-1.24) |  |
| + | + | 196 | 7.7 (4.7) | 69 (35.2) | 1.18 (0.92-1.50) | 1.20 (0.94-1.53) |  |
| + | - | 233 | 7.9 (4.8) | 92 (39.5) | **1.29 (1.02-1.61)** | **1.26 (1.01-1.58)** | -0.05 (-0.37; 0.26) |
| EDSS 4 | | | | | | |  |
| Smoker | DRB1*15:01 | N | Years (SD) | Outcome (%) | HR (95% CI)^1^ | HR (95% CI)^2^ |  |
| - | + | 614 | 9.7 (4.2) | 95 (15.5) | 1.0 (reference) | 1.0 (reference) |  |
| - | - | 748 | 9.9 (4.4) | 131 (17.5) | 1.06 (0.83-1.35) | 1.08 (0.85-1.39) |  |
| + | + | 196 | 9.4 (4.4) | 36 (18.4) | 1.36 (0.98-1.91) | **1.41 (1.00-1.98)** |  |
| + | - | 233 | 9.6 (4.2) | 46 (19.7) | **1.36 (0.99-1.88)** | **1.37 (1.00-1.89)** | -0.09 (-0.55; 0.36) |

HLA=human leukocyte antigen; HR=hazard ratio; AP=attributable proportion due to interaction; CI=confidence interval; SD=standard deviation, CDW=clinical disease worsening; EDSS=expanded disability status scale. ^1^adjusted for sex and age at baseline; ^2^adjusted for sex, age at baseline, ancestry; past infectious mononucleosis; calendar year of diagnosis, disease phenotype; baseline EDSS, disease duration at baseline, proportion of follow-up spent on disease-modifying therapy; educational attainment; body mass index; alcohol consumption; fish consumption; sun exposure habits; and physical activity.

eFigure 1. Flow chart


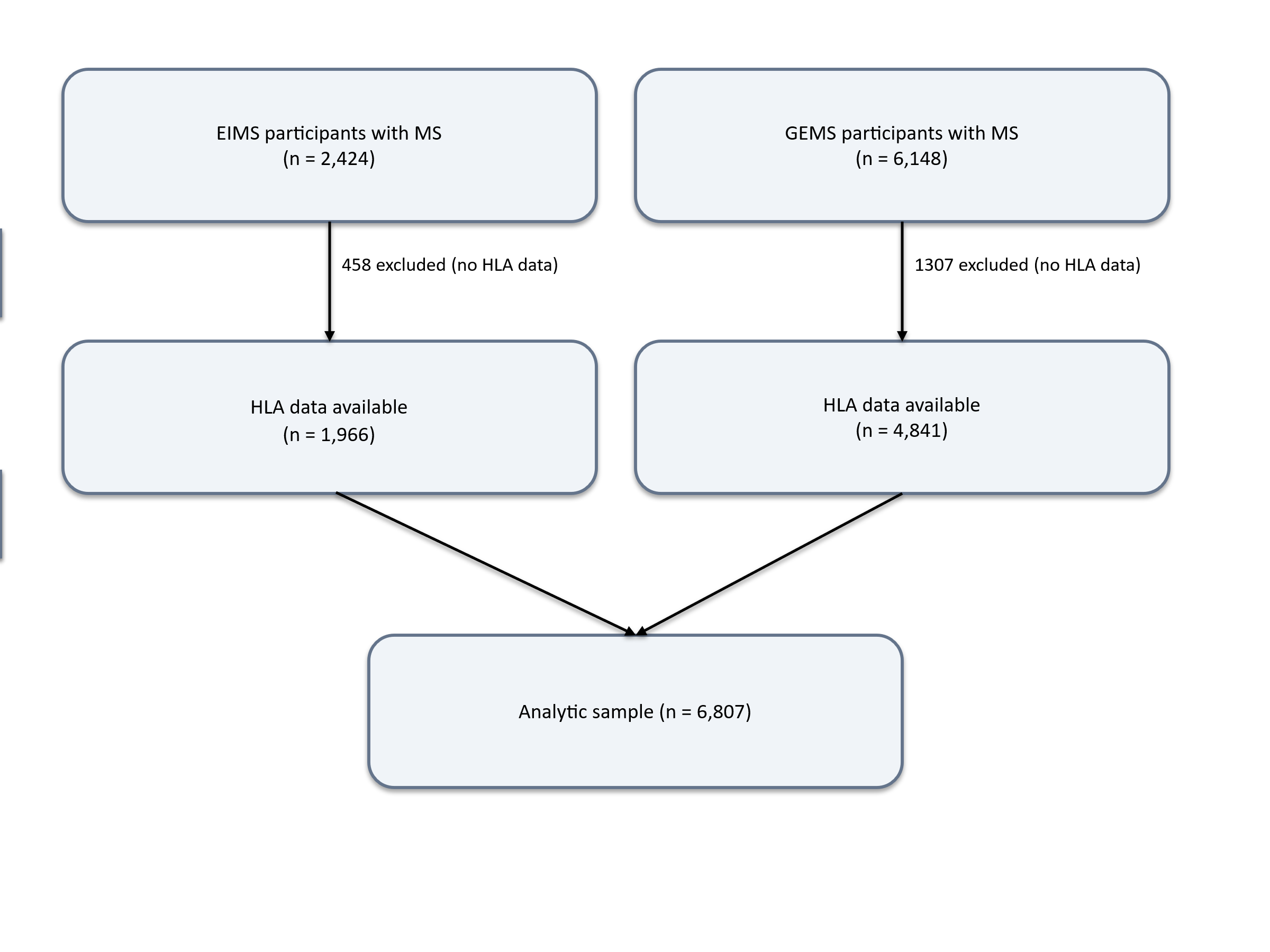


eFigure 2. Hazard ratios for disability progression by HLA-DRB1*15:01 and smoking status.


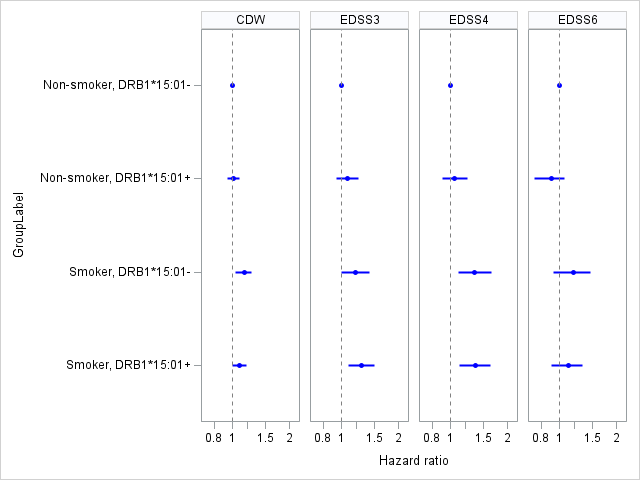


HLA=human leukocyte antigen; EDSS=expanded disability status scale; CDW=confirmed disability worsening; DMT=disease-modifying therapy. Forest plot showing hazard ratios and 95% confidence intervals for CDW and EDSS 3, 4, and 6. The reference group is HLA-DRB1*15:01 negative non-smokers. Adjusted for sex, ancestry, age at baseline, past infectious mononucleosis; calendar year of diagnosis, disease phenotype; baseline EDSS, disease duration at baseline, and proportion of follow-up spent on disease-modifying therapy.

eFigure 3. Time to EDSS 4, by HLA-DRB1*15:01 and smoking status


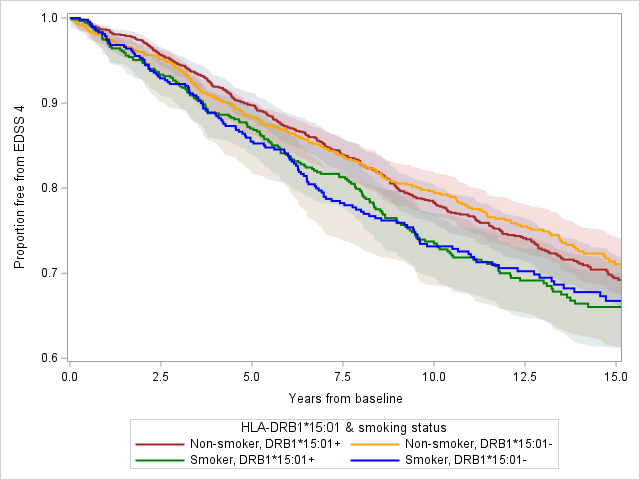


EDSS=expanded disability status scale; HLA=human leukocyte antigen. Curves represent Kaplan-Meier survival estimates up to 15 years post-diagnosis. Groups are defined by smoking status at diagnosis (smokers vs non-smoker) and presence/absence of the HLA-DRB1*15:01 allele. Shaded areas represent 95% confidence intervals. Numbers at risk at 0, 5, 10 and 15 years were: non-smoker, DRB1*15:01+: 1457, 1179, 798, 334; non-smoker, DRB1*15:01-: 1119, 890, 611, 213; smoker, DRB1*15:01+: 590, 483, 321, 144; smoker, DRB1*15:01-: 489, 370, 250, 120.
